# Metropolitan Hospital Sunday Fund

**Published:** 1911-03-18

**Authors:** 


### METROPOLITAN HOSPITAL SUNDAY FUND.

### SUNDAY CINEMATOGRAPH SHOWS AND HOSPITALS.

A meeting of the Council of the Fund was held on Friday, March 10, under the presidency of the Lord Mayor, to receive recommendations of the Finance Committee as to the appointment of new trustees, and of the General Purposes Committee in the matter of Sunday cinematograph shows. Mr. Hale said it would be remembered that when the Council was started three gentlemen were asked to act as trustees for the fund—the Lord Mayor, the Governor of the Bank of England, and the Chamberlain for the time being. That had worked very fairly, and thanks were due to those gentlemen for what they had done; but it was not now found to be very practicable, chiefly for two reasons. The first was, that the continued transferring of the securities to each succeeding Lord Mayor and Governor of the Bank of England caused much trouble and expense. It had been thought expedient by many members of the Council that it should be altered, and the Finance Committee recommended that the application should be made before the court, and that four gentlemen should be appointed as trustees.

Sir Richard Martin moved : That the recommendation of the Finance Committee be, and is hereby, approved and adopted, and that, in accordance therewith, Mr. John Hampton Hale, Mr. John Henry Buxton, Sir John Charles Bell, Bart., and Sir Charles Ernest Tritton, Bart., be and are hereby appointed trustees to the securities, subject to the approval of the court. This was carried unanimously.

### Sunday Cinematograph Shows in Aid of Hospitals.

The Ven. Archdeacon of London (Archdeacon Sinclair) brought forward the following resolution :—

That the Council has fully considered the recommendations of the General Purposes Committee on the subject of Sunday cinematograph entertainments run by hospitals, as well as the proposal of certain places of worship to ear-mark their collections for hospitals which do not participate in the proceeds of such shows, and while not expressing any opinion upon the expediency or otherwise of Sunday entertainments as such, disapproves of hospitals receiving grants from this Fund (which consists mainly of Church collections), being concerned in any movement which is contrary to the spirit of its supporters.

The Council further resolves that in the event of the collections for the Fund suffering any diminution which may be fairly attributable to the continuance of these entertainments, as aforesaid, it shall be a recommendation to the Committee of Distribution to take the same into consideration when making awards for the year.

It is ordered that a copy of this resolution be forwarded to the Committee of Management of each of the hospitals concerned.

In supporting it, he said the matter was one of some delicacy; but it was clearly defined and in strict limits. It was simply that three or four powerful leading churches in the Metropolitan area, both Church of England and Nonconformist, were so displeased at the method by which four of the hospitals were raising part of their funds that they asked to ear-mark their contributions to hospitals which did not employ that method. That would, in their opinion, break up the Hospital Sunday Fund, and many of the congregations would follow the suggested precedent. The method to which objections were raised was the holding of shows at electric theatres on Sunday afternoons for the hospitals. The law at present was very clear on the subject of opening places of public amusement on Sundays for profit. Those who catered for public amusements, the employees of theatres and music-halls, were grateful for that prohibition, and they had petitioned again and again against any relaxation of that law. Electric theatres, which were a new institution and were springing up in large numbers all over London, had found a way of getting round the law. In recent years the licensing of those shows had been placed in the hands of the London County Council, and the Act did not repeat the caution against Sunday trading; therefore those places of entertainment had obtained the leave of the London County Council to open on Sunday on the condition that the show was run on that day by a charitable society. The proprietors could charge what they liked for expenses, and by charging a heavy amount for expenses the proprietors could make a good profit. Four hospitals, including the London and the Evelina, made substantial gains. It had been said that the London Hospital made £1,300 a year in that way.

### Objections to Sunday Shows.

There were two sets of people who objected to those electric theatres holding the Sunday entertainments : firstly, those who said they regarded the proceedings as a serious evasion of the law, and as likely to have an effect on the enormous number of caterers for public amusement who required their day of rest. Secondly, there were those who regarded it as an infringement of that principle of a day of rest by which the customs of this country had been regulated. The churches were not in the least jealous on account of the attendance at their places of worship, for, happily, the churches which objected were always full. With regard to neither of those objections did he express any opinion, nor did the Committee. When some of the most powerful and influential congregations in London objected so strongly to the method, and wished to ear-mark their contributions so that those contributions should go to hospitals which did not hold such shows, it was felt that the Council should do what it could to protect the scruples of those congregations and preserve the integrity and the harmony of the Hospital Sunday Fund, which had been so long and so happily maintained. They did not propose that the grants to those hospitals should be diminished by the sums which were obtained by such shows, and they did not express condemnation of the electric-theatre shows themselves, but they wished to dissociate themselves from that method of collecting money for the hospitals. Some people said money would be collected from such shows on Sunday just the same, and it would be given to all sorts of charities, some of which were of a less solid utility than the hospitals. The Committee took a wider view than that. If the method was such that it caused genuine offence to the most influential congregations, the Committee must take whatever opportunity it had to prevent such offence. That appeared to be the business of the Council of the Fund, for the Fund was not raised in any ordinary way; it was not like an ordinary business concern which had no direct relation to the consciences of the people. The collections were made from religious people scattered throughout the Metropolitan area, and they were given on religious grounds, by religious persons, for a religious object. The Committee had no complaint against the London Hospital, or any other hospital; it sympathised with the responsibility of raising annually enormous sums which were necessary, and it was grateful for the energy and zeal displayed for so many years by the Treasurer of the London Hospital. By the recent methods pursued the funds of the Hospital Sunday Fund had been placed in jeopardy. The Committee believed that the action it recommended would satisfy the scruples which had been raised.

Lord Cheylesmore's Support.

The Right Hon. Lord Cheylesmore, K.C.V.O., seconded the resolution. He had nothing to say against the shows, as he had run two of them himself, but that was different from Sunday shows. The bulk of the money for the Hospital Sunday Fund was raised through the advocacy of clergymen, and the Council should stick to the clergy and their congregations. He would correct the Archdeacon and point out that the proprietors of the shows could not charge what they liked. The proprietor of a show had to satisfy the Committee of the London County Council what they were getting for it on each particular Sunday afternoon, and they were not entitled to show any profit. In future there would be some alteration, and the London County Council would limit the number of shows which could be run by any charity.

Mr. Sydney Holland's Appeal for Toleration.

The Hon. Sydney Holland moved an amendment, as follows: "That the Council take no action in this matter." On looking round at the audience, he felt no doubt that he would be beaten that day, but he was appealing to a larger audience than was gathered in the room, among whom there would doubtless be an equally strong feeling on the other side. He did not think anyone could accuse him of being in any way hostile to the Fund. The greatest friend he ever had in the world, with whom he had the opportunity of discussing the Fund, and whose executor he was, left no less than £600,000 to the Fund. He had spoken for the Fund in pulpits, and had even had to face Mr. Stephen Coleridge for it at a Mansion House meeting! But to-day he regretted he was obliged to differ from his colleagues on the Council. The London Hospital receive between £1,200 and £1,400 a year from Sunday shows. Therefore one would have thought that before such a decision was come to by the General Purposes Committee he might have been heard. He was asked to attend a meeting of the Committee, and he got there a few minutes before the time stipulated, but found that the whole matter had already been settled, the resolutions having been in type beforehand. The shows were licensed by the London County Council on the condition that the proceeds, less certain audited deductions, went to the charities.

Churches and Sunday.

Certain clergymen objected to the shows and desired to allocate their subscriptions to hospitals which did not take money from such shows, and in that his friend Prebendary Gurdon took a leading line. But he (Mr. Holland) was to be seconded by the Rev. Mr. Gamble, the collection at whose church was the third in magnitude—Holy Trinity, Sloane Street. Not only would many clergymen differ, but a large number of the public would differ from what he regarded as a very narrow-minded policy. If the objectors allocated their subscriptions to the hospitals which did not take money from that Fund, then it was open for others to allocate theirs to hospitals which did take money from the Fund. But it was not those who ran such shows who disturbed the peace of the Fund. It was the Archdeacon who had thrown the bombshell. He believed Mr. Gurdon's better sense would prevail in time, especially if he found, as he probably would, that many of his congregation were against him. The resolution meant that the money collected by the Sunday Fund would not go to the hospital which was best managed or deserved it most, but to those hospitals whose managers were most pliable and obedient to the wishes of these dictators; and it did not seem to him a very admirable way of enforcing views on what must be a very debatable question. The principle of the Fund had always been that it would not interfere in the way a hospital got its money, but would and did interfere in the way in which it spent it. There was nothing in the shows to prevent people attending church if they wished to. What would be the next step? Perhaps many clergymen who were strong teetotalers would be saying they would allocate their subscriptions to hospitals where no intoxicants were given out, or to one which had no brewers on the Board, or they would be told they ought not to take the vast sum which they got from Mr. Sam. Lewis because he raised his money by money-lending. Or they might be told by another set of clergymen that no money which was made by speculation should be received. If that sort of thing was once begun there would be no end to it. The great point was that the Sunday Fund did not take money from those shows, therefore it was nothing to do with the Fund how that money was raised.

Raising Money for Hospitals.

If money were raised for a hospital in some scandalous way, then the Committee might well step in and say they would give nothing to such a hospital. But nothing of the sort could be said against these shows. He had attended them; they were morbidly sentimental and educationally dull, though he admitted he once saw a boxing contest portrayed, which might be regarded as very immoral. But the shows were quiet and orderly, and did not take place in the morning, so that they did not prevent people going to church. It was much better to go to such shows than to walk about the streets, or spend the time in public-houses, and the money went to the best of charities. Could that be wrong? It almost purged the offence, if such there were, when the object was so good. With regard to Sunday observance, it was almost impossible, if not indeed impertinent, for one man to tell another what was right and what was wrong, because it was a matter of opinion and of thought. Personally he was rather a Sabbatarian. He was a pillar of the Church—not a caterpillar—he did not play golf on Sunday—there was an age-limit to golf, so probably members of the Council did not play it, though their grandchildren did. But he would not hold up his hands and thank God he was not as other men because somebody else played golf on Sunday. One Roman Catholic church took the whole of the proceeds of the cinematograph shows for itself, and Lord Cheylesmore admittedly ran two shows himself, but objected to these for the hospitals. But the action would not stop the shows; it would divert the money from the best of charities to other charities, and so the suggestion was a monstrous one. Why was there no protest against the enjoyments of the rich? They had the Albert Hall and Queen's Hall concerts, all of which were paid for; but these humble shows for the poor were to be stopped, or at least the money raised by them was not to be given to the hospitals. He felt that what he was saying would have very little effect at that meeting, but perhaps the effect would be greater in the larger constituency. He had not had time to go round and see people, as others had done; but he was aware that most of those present had already decided against him. But he had to bear the burden of collecting for the London Hospital, of which only £20,000 was from endowments. The burden was almost intolerable, and if the Council by its action made the burden more intolerable, he invited it to take over the responsibility of that task. He urged his hearers to pause before taking this action.

A Clergyman Supports the Amendment.

The Rev. Mr. Gamble (Holy Trinity, Sloane Street) seconded the amendment. He said the motion which had been proposed was candid, and it was the result of the Council having given way at the pressure of half-a-dozen congregations. Was there anybody present who seriously took up the position that all amusements and recreations on Sunday were wrong? (No.) Therefore the only question which remained was as to how far they were expedient. There were many Sunday recreations which he admitted might be considered inexpedient; for instance, in the case of the man who worked his chauffeur seven days a week, and the man who, having all the days of the week to himself, devoted his Sunday to golf, thus keeping caddies away from Sunday morning church. But he could not imagine any amusements less liable to objection than those under discussion. The greater part of the people of London lived in very poor and mean tenements, and they could not be expected to spend the whole of their Sunday at home. On the Continent there were cafés and beer-gardens, where they could meet for sensible recreation in a rational manner, without being tempted to excessive drinking. But in this country Puritanism aimed not at the improvement of the public-house, but at its destruction, or, failing that, its degradation. It was not fair to suppose that the people of London spent all their time in either church or their own homes, or in walking about the streets. The shows in question did not commence until four, they had been described as even dull, and they did not prevent people who wished to do so from attending public worship; and finally, those who carried on the shows were not overworked.

Another Plea for Toleration.

He agreed with Mr. Sydney Holland in believing that in taking the present action they were entering upon a very dangerous field. Once enter upon investigations as to the sources from which money came from the public to hospitals, and the consequences might be very awkward. If brewers were to give large sums of money to hospitals, fanatical teetotalers would describe it as blood money, which should not be touched: there was no limit to the forces of fanaticism when once they were let loose. As to the threat to ear-mark contributions so that they should go only to certain hospitals, two could play at that game. He did not say he would do so himself, but he would take the advice of his congregation on the subject. Such a warfare would be very detrimental to the Fund, and he asked the Council not to take such a dangerous step, but to leave it to the consciences of those who managed hospitals, whence they received their money. To praise Mr. Sydney Holland's work for the London Hospital would be an impertinence, and he thought that gentleman might well be trusted, as could also the managers of other hospitals.

Sir Alfred Pearce Gould, speaking in favour of the original resolution, appealed to Mr. Holland not to press his amendment. Everybody sympathised with Mr. Holland's great task in connection with the London Hospital, but he raised the money by means of voluntary contributions. There was nothing voluntary about the money received from cinematograph shows. The hospitals of London were great voluntary institutions: once admit that the hospitals could be supported by other means, and there would be rate-aided and rate-supported hospitals. Why did not Mr. Holland open those shows for the purpose on the other days of the week? (Mr. Holland: That is a good idea; I will do so.)

Invidious Distinctions Deprecated.

The Rev. C. H. Grundy urged the necessity of the mind being cleared of cant in the matter. Cinematograph shows gave the greatest amount of pleasure with the smallest expenditure of labour. He admitted that care might have been taken to portray religious subjects at the shows, but he asked the Council to have no hand in the passing of the resolution. He objected to Archdeacon Sinclair's use of the word "influential" in his address; it was the old story of the Church of England: they could not afford to offend the West End vicar with his enormous congregation and large collection. His own congregation numbered about 1,100, and they would not look at such a resolution as that now proposed.

On the amendment being put, ten voted for and nineteen against.

"The previous question" was then moved, and lost.

The Resolution Referred Back.

Mr. Deputy Wallace moved that the question be referred back to the General Purposes Committee, so that Mr. Holland might have the opportunity of discussing it before them. It was, he contended, a very serious matter which was now before the Council, and nothing should be done which could result in imperilling the success of the Fund, which was accomplishing so much good. Mr. Holland's work fully entitled him to be heard by the General Purposes Committee. It was serious that that gentleman should have found, when he attended the meeting of the General Purposes Committee, that the matter for which he attended had already been disposed of.

The Rev. Mr. Pierce seconded Mr. Wallace's proposition.

The Lord Mayor agreed that what Mr. Holland said concerning the meeting of the General Purposes Committee was technically correct; but later, in the presence of Mr. Holland, the subject was reopened and discussed at considerable length. But nothing new was advanced, and the previous decision was not altered.

The Archdeacon of London agreed that no new argument was advanced by Mr. Holland at the meeting in question. He had no objection to taking the matter back again.

Mr. Sydney Holland said he did not see any good in referring the question back, as the Committee had already made up its mind.

Several speakers urged Mr. Holland to agree to the Archdeacon's offer, and on being put to the vote, nineteen were in favour of referring back, and five against.

National Anti-Vivisection Hospital.

The Secretary stated that he had received a number of letters from the National Anti-Vivisection Hospital; and it was agreed to appoint Mr. Hale and Mr. Norman a sub-committee to go through the letters and report the result to the Council. He had also received letters with copies of resolutions recently passed at Caxton Hall protesting against subsidising London hospitals for the medical treatment of school children from the Independent Labour Committee for Promoting the Welfare of Children.

